# A nationwide cohort study on incidence and mortality associated with extracranial vascular malformations

**DOI:** 10.1038/s41598-023-41278-z

**Published:** 2023-08-25

**Authors:** Jeong Yeop Ryu, Yong June Chang, Joon Seok Lee, Kang Young Choi, Jung Dug Yang, Seok-Jong Lee, Jongmin Lee, Seung Huh, Ji Yoon Kim, Ho Yun Chung

**Affiliations:** 1 Department of Plastic and Reconstructive Surgery, School of Medicine, Kyungpook National University, 680 Gukchaebosanro, Jung-gu, Daegu, 41405 Republic of Korea; 2 Department of Dermatology, School of Medicine, Kyungpook National University, Daegu, Republic of Korea; 3 Department of Radiology, School of Medicine, Kyungpook National University, Daegu, Republic of Korea; 4 Department of Surgery, School of Medicine, Kyungpook National University, Daegu, Republic of Korea; 5 Department of Pediatrics, School of Medicine, Kyungpook National University, Daegu, Republic of Korea; 6 Cell and Matrix Research Institute, School of Medicine, Kyungpook National University, Daegu, Republic of Korea

**Keywords:** Epidemiology, Outcomes research, Vascular diseases

## Abstract

Extracranial vascular malformations are abnormal formations of blood vessels located outside the brain (extracranial) that develop during fetal development. They are caused by errors in the formation of blood vessels in the embryo and can affect various parts of the body, such as the head, neck, face, and other regions. Some malformations may be asymptomatic and only require monitoring, while others may cause significant health issues or cosmetic concerns and may need medical intervention. There are very few studies have investigated the nationwide incidence and quantitative mortality of vascular malformations in terms of their subtypes. Thus, this study aimed to determine the nationwide incidence and mortality associated with vascular malformations. This nationwide population-based study evaluated 70,517 patients with vascular malformations from 2008 to 2021. We evaluated the incidence and mortality associated with each subtype of vascular malformation. Furthermore, Cox regression analysis was used to evaluate the association between vascular malformation and mortality. The annual incidence (per 100,000 population) of overall vascular, venous, capillary, arteriovenous, and lymphatic malformations was 9.85, 1.48, 2.31, 0.24, and 5.82 cases, respectively. Patients with vascular malformations, except those with venous malformations, had higher mortality than the matched controls. Moreover, among the vascular malformation subgroups, the adjusted hazard ratio of mortality was the highest for arteriovenous malformations. This study revealed that the overall annual incidence of vascular malformations was 9.85 cases per 100,000 population in Korea from 2008 to 2021. The mortality of the matched general population was lower than that of patients with vascular malformations, except for those with venous malformations. Additionally, the adjusted hazard ratio for mortality associated with arteriovenous malformations was the highest among the vascular malformation subgroups.

## Introduction

According to the International Society for the Study of Vascular Anomalies classification, extracranial vascular malformations are a subtype of extracranial vascular anomalies. Simple extracranial vascular malformations are classified into the subtypes of venous malformations (VMs), capillary malformations (CMs), arteriovenous malformations (AVMs), and lymphatic malformations (LMs). When these subtypes are mixed, the malformation is classified as a combined malformation^1^.

Among the extracranial vascular malformations, VMs are caused by abnormalities in vascular morphogenesis and are characterized by slow-flow extracranial vascular malformations. Notably, most VMs appear as isolated lesions, whereas some appear as multifocal or diffuse lesions. Moreover, VMs most frequently occur in the head and neck regions, and they clinically manifest in the form of bluish or purple subcutaneous nodules. The natural history of VMs indicates that the corresponding lesions appear at birth; however, parents of patients with VMs may notice these lesions when the child has grown. Moreover, VMs grow slowly and do not regress spontaneously. Therefore, in cases of lesions present in the deep tissue, it may be difficult to notice the lesion until the appearance of pain or swelling^2^. VMs are diagnosed by taking the history of the patient and performing physical examinations, Doppler ultrasonography, and magnetic resonance imaging (MRI). Furthermore, VMs are mainly treated by sclerotherapy and/or surgical excision^3^. Regarding their incidence, one study reported approximately 1 case of VM in 2000–5000 births, and another study reported 1–5 cases in 10,000 births^4,5^.

CMs were formerly called port-wine stains, which are slow-flow extracranial vascular malformations associated with enlarged capillaries and venules in the skin and/or mucous membranes. The colors of these lesions often appear significantly light in the first few months of life; however, as the child ages, the color gradually darkens owing to the concentration of hemoglobin^6^. Moreover, like VMs, these lesions appear most frequently in the head and neck regions, and they invade the lip, gingiva, and oral mucosa^7^. Several treatments have been reported for CMs; however, laser therapy has been recognized as the gold standard. Furthermore, the decision about the treatment of CMs is recommended to be made by comprehensively considering the size, depth, and location of the lesion, but surgical treatment may be required if the soft tissue or bony hypertrophy is present^8,9^. Some studies have suggested that CMs are present in 0.3–9.1% of the population; however, only a few studies have reported the epidemiology of CMs^7,10,11^.

AVMs are caused by the absence of a capillary bed, resulting in the shunting of blood from the artery to the vein through the nidus. Notably, nidus is an abnormal channel bridging the feeding artery to the draining veins. The occurrence of AVMs can be attributed to five causes: endothelial cell remodeling, biomechanical activation, hormonal stimulation, extracellular matrix dysregulation, and pericyte dysfunction. Furthermore, they are characterized by high-flow extracranial vascular malformations, which are the most devastating vascular anomalies. Notably, patients with AVMs experience pain, ulcers, bleeding, and tissue destruction. In cases of severe progression of symptoms, such patients may experience high-output cardiac failure^12^. The treatment of AVMs is determined by the size, depth, and shape of the lesion; risk of bleeding; and site of occurrence. The treatment modalities include sclerotherapy using ethanol and bleomycin and surgical resection with or without embolization^13^. In the relevant literature, some studies have reported the incidence of cerebral AVMs. One study suggested that the rate of detection of cerebral AVMs was 1.21 per 100,000 person-years^14^. Another study reported that the prevalence of cerebral AVMs was 15–18 per 100,000 adults^15^. However, to the best of our knowledge, only a few reports have indicated the incidence or prevalence of extracranial AVMs.

LM comprises lymphatic fluid-filled vesicles and is the modern term for the antiquated lymphangioma. LMs can be caused by abnormal development, particularly related to embryological disorders of the lymphatic system. Moreover, they are classified by the size of the malformed lymphatic channel as follows: macrocystic, microcystic, or combined. Additionally, LMs may be localized or involve large areas throughout the body^16–18^. Furthermore, although these lesions can occur anywhere on the body, they can cause facial asymmetry when occurring on the face. In particular, in cases of intraorbital involvement, ocular dystopia, exophthalmia, orbital proptosis, and visual loss may occur. Treatment modalities for LMs include sclerotherapy and surgical excision. The decision about the treatment is determined by a comprehensive evaluation according to the size, depth, shape, and location of the lesion. Regarding the epidemiology of LMs, one study reported that the incidence of LMs is 1.2–2.8% per 100,000 hospital admissions, whereas another study reported the incidence is 2.8% per 100,000 hospital admissions^19,20^. In the relevant literature, the reported incidences of extracranial vascular malformations have been very diverse to date. In addition, some studies have reported the incidence in terms of population, whereas others have reported it in terms of birth. This variety in the number and methodology of expression reflects the difficulty in studying the incidence of extracranial vascular malformations.

Few studies have reported the incidence or prevalence of overall extracranial vascular malformations. Kennedy WP et al. reported the overall incidence of extracranial vascular malformations as 1.08% ranging from 0.83 to 4.5% based on comprehensive review of 238 studies on the world literature reporting more than 20 million births. Tasnadi et al. reported overall incidence of the extracranial vascular malformations in 1.2% (43 out of 3573) based on a study carried on 3573 three-year-old children^21,22^.

Although most extracranial vascular malformations are benign, some slow-flow malformations may be associated with hematological disorders. Moreover, high-flow malformations, including AVMs, can cause heart dysfunction, which may lead to high mortality. However, although some studies have reported on mortality associated with extracranial vascular malformations, these studies were based on hospital data and their sample size was small^23,24^. Thus, to better compare the prognosis between the subtypes of extracranial vascular malformation, further nationwide studies are needed for mortality associated with extracranial vascular malformations.

The aim of this study was to investigated the nationally representative incidence and mortality associated with each vascular malformation subtype based on the Korean National Health Insurance Service (NHIS) claims database.

## Methods

### Ethical approval

This study was approved by the Institutional Review Board of Kyungpook National University Hospital (IRB No. KNUH 2021-02-011) and was performed in accordance with the principles of the Declaration of Helsinki. All personal information was anonymized. Disclosure and sharing of anonymized health insurance data were guaranteed by Korean law, and there was no reason to presume that participants refuse to consent. Because all data was anonymized, the risk of the study due to the waiver of consent was extremely low. Therefore, informed consents for participant’s waiver were obtained from Institutional Review Board of Kyungpook National University Hospital. Prior to data sharing from National Health Insurance Sharing Service (NHISS), our protocol for data analysis was approved by Deliberation Committee of NHISS in Korea (No. REQ202101156-002).

### Data source

This study was conducted using the Korean NHIS-National Health Information Database (NHIS-NHID). This database is operated by NHIS and includes data of the entire Korean population (over 50 million individuals)^25,26^. Notably, health insurance services of Koreans were covered under the mandatory NHIS system. Moreover, NHIS-NHID contains comprehensive information about Korean patients, including demographic characteristics, history of inpatient and outpatient care, main diagnosis and comorbidities based on the International Classification of Disease 10th revision (ICD-10) codes, and history of diagnostic or therapeutic procedures and surgical treatments^27^.

### Study population and outcomes

**Figure 1:**
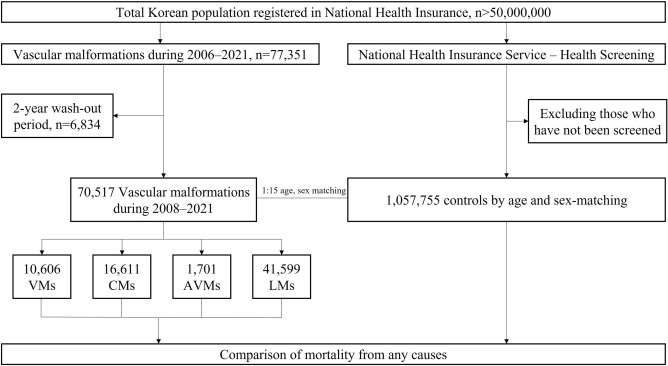
Flowchart of the study. *VMs* venous malformations, *CMs* capillary malformations, *AVMs* arteriovenous malformations, *LMs* lymphatic malformations.

Patients with extracranial vascular malformations who were registered in NHIS-NHID between January 2006 and December 2021 were included in the study (Fig. 1). These patients were identified based on the ICD-10 codes. The cases of VMs, CMs, and LMs were defined as Q279, Q273 or Q825, and I899 or D181, respectively. Furthermore, the cases of AVMs were defined as Q273 and classified into those who underwent sclerotherapy (M177, O021) or embolization (M16, M66). To prevent the inclusion of prevalent cases from the database and only select newly diagnosed patients, a 2-year washout (2006–2007) period was applied in the analysis.

The primary outcomes of this study were the incidence of extracranial vascular malformations and mortality due to any cause during 2008–2021 in terms of age, sex, and vascular malformation subtype. The incidence of extracranial vascular malformations was defined by a new diagnosis of vascular malformation. The annual incidence was calculated by dividing the total incidence by the total population of the corresponding year. Average incidences were calculated by dividing the total number of cases by the overall population in each age- and sex-specific group and averaging the data from 2008 to 2021^28^. Mortality was identified according to the demographic information obtained from NHIS-NHID. Further, data on the date of death were collected for all cases.

### Diagnostic accuracy

Overall, 538 patients with extracranial vascular malformations who visited Kyungpook National University Hospital from 2006 to 2021 were evaluated by reviewing medical records. Two plastic surgeons performed the diagnostic validation. Among patients with histologically confirmed extracranial vascular malformations, the percentage of those who met the diagnostic criteria was defined as sensitivity. In contrast, among patients without extracranial vascular malformations, the percentage of those who did not meet the diagnostic criteria was defined as specificity. The sensitivities for diagnostic codes of VMs, CMs, AVMs, and LMs were 94.06%, 92.08%, 89.11%, and 93.07%, respectively. The specificities for diagnostic codes of VMs, CMs, AVMs, and LMs were 92.16%, 92.71%, 91.18%, and 93.14%, respectively.

### Statistical analysis

The trends of incidences of all vascular malformation subtypes were evaluated using Poisson regression. Further, the mortality of patients was assessed by calculating person-years for the study population and compared between patients with extracranial vascular malformations and the general population never diagnosed with extracranial vascular malformations. For each patient with extracranial vascular malformations, 15 people without extracranial vascular malformations on the index date from the general population in the NHIS-Health Screening were matched by the year of birth and sex^29^. To define control cohort, we divided the year of birth of all patients with extracranial vascular malformations into 5-year increments. In addition, after sex was divided into male and female, a 15-fold general population was randomly selected for the year and sex of each patient. Socioeconomic indices or regional status were not reflected.

Risk factors for mortality among patients with extracranial vascular malformations were evaluated using Cox proportional hazard models. The results were reported in terms of hazard ratios (HRs) and 95% confidence intervals (CIs) for mortality. Further, these models were adjusted for potential confounding factors, including birth year and sex^26^. All statistical tests were evaluated using two-tailed 95% CIs, and a *P* value of < 0.05 was considered statistically significant. All analyses were performed using STATA software STATA/MP2 (version 17.0; StataCorp, College Station, TX).

## Results

### Distribution and incidence rate of extracranial vascular malformations

Table 1 shows the annual incidences of extracranial vascular malformations in Korea from 2008 to 2021. In total, 70,517 patients with extracranial vascular malformations were identified during this period, with an annual incidence of 9.85 cases per 100,000 population. Notably, the annual incidence was higher among females than among males (11.37 vs. 8.33 cases per 100,000 population). Moreover, female patients had a higher incidence of extracranial vascular malformations than male patients in all subtypes, with the greatest proportion observed for AVMs (65.31%) (Table 1). The most diagnosed subtype was LMs (41,599 patients, 59.0%), followed by CMs (16,611 patients, 23.6%), VMs (10,606 patients, 15.0%), and AVMs (1701 patients, 2.4%). The annual incidences (per 100,000 population) for LMs, CMs, VMs, and AVMs were 5.82, 2.31, 1.48, and 0.24, respectively. Poisson regression performed to determine the trend in annual incidence did not reveal any trends in the incidence of extracranial vascular malformations or incidence by subtype (Table 2).

**Table 1:** Incidence of vascular malformations from 2008 to 2021 in Korea.

	Total	VMs	CMs	AVMs	LMs
Number of patients (n)	70,517	10,606	16,611	1701	41,599
Percentage of all vascular malformations	100.0	15.0	23.6	2.4	59.0
Incidence (/100,000/year) (95% CI)	9.85 (9.06–10.64)	1.48 (1.32–1.63)	2.31 (2.02–2.60)	0.24 (0.22–0.26)	5.82 (4.94–6.71)
Number of females (%)	40,716 (57.74)	5,988 (56.46)	9,214 (55.47)	1,111 (65.31)	24,403 (58.66)
IR males (/100,000/year) (95% CI)	8.33 (7.52–9.14)	1.29 (1.15–1.43)	2.06 (1.86–2.27)	0.16 (0.15–0.18)	4.82 (3.88–5.75)
IR females (/100,000/year) (95% CI)	11.37 (10.53–12.20)	1.67 (1.49–1.85)	2.56 (2.18–2.95)	0.31 (0.28–0.34)	6.83 (5.97–7.69)
Mean age at diagnosis (years) (95% CI)	46.14 (45.98–46.30)	32.69 (32.26–33.13)	41.17 (40.85–41.49)	40.44 (39.55–41.33)	51.78 (51.59–51.97)

*CI* confidence interval, *VMs* venous malformations, *CMs* capillary malformations, *AVMs* arteriovenous malformations, *LMs* lymphatic malformations, *IR* incidence rate.

**Table 2:** Poisson regression analysis for annual incidence of vascular malformations from 2008 to 2021 in Korea.

	Coefficient (per year) (95% CI)	Incidence rate ratio (per year) (95% CI)	*P* value
Total, all	0.0400 (− 0.0549 to 0.1349)	1.0408 (0.9466–1.1445)	0.408
Total, male	0.0245 (− 0.0802 to 0.1292)	1.0248 (0.923–1.1379)	0.646
Total, female	0.0506 (− 0.0369 to 0.1381)	1.0519 (0.9638–1.1481)	0.257
VMs, all	0.0452 (− 0.1957 to 0.2861)	1.0462 (0.8222–1.3313)	0.713
VMs, male	0.0324 (− 0.2282 to 0.2931)	1.033 (0.796–1.3406)	0.807
VMs, female	0.0546 (− 0.1708 to 0.2799)	1.0561 (0.843–1.323)	0.635
CMs, all	0.0383 (− 0.1551 to 0.2318)	1.0391 (0.8563–1.2608)	0.698
CMs, male	0.0199 (− 0.1882 to 0.2279)	1.0201 (0.8284–1.256)	0.852
CMs, female	0.0521 (− 0.1296 to 0.2338)	1.0535 (0.8785–1.2634)	0.574
AVMs, all	0.0307 (− 0.5802 to 0.6415)	1.0311 (0.5598–1.8993)	0.922
AVMs, male	0.0344 (− 0.6967 to 0.7656)	1.035 (0.4982–2.1502)	0.926
AVMs, female	0.0286 (− 0.5061 to 0.5634)	1.0291 (0.6029–1.7566)	0.916
LMs, all	0.0321 (− 0.0942 to 0.1584)	1.0326 (0.9101–1.1716)	0.619
LMs, male	0.0156 (− 0.1257 to 0.1568)	1.0157 (0.8819–1.1698)	0.829
LMs, female	0.0431 (− 0.0722 to 0.1584)	1.0441 (0.9304–1.1717)	0.464

*CI* confidence interval, *VMs* venous malformations, *CMs* capillary malformations, *AVMs* arteriovenous malformations, *LMs* lymphatic malformations.

### Age and sex distribution

**Figure 2:**
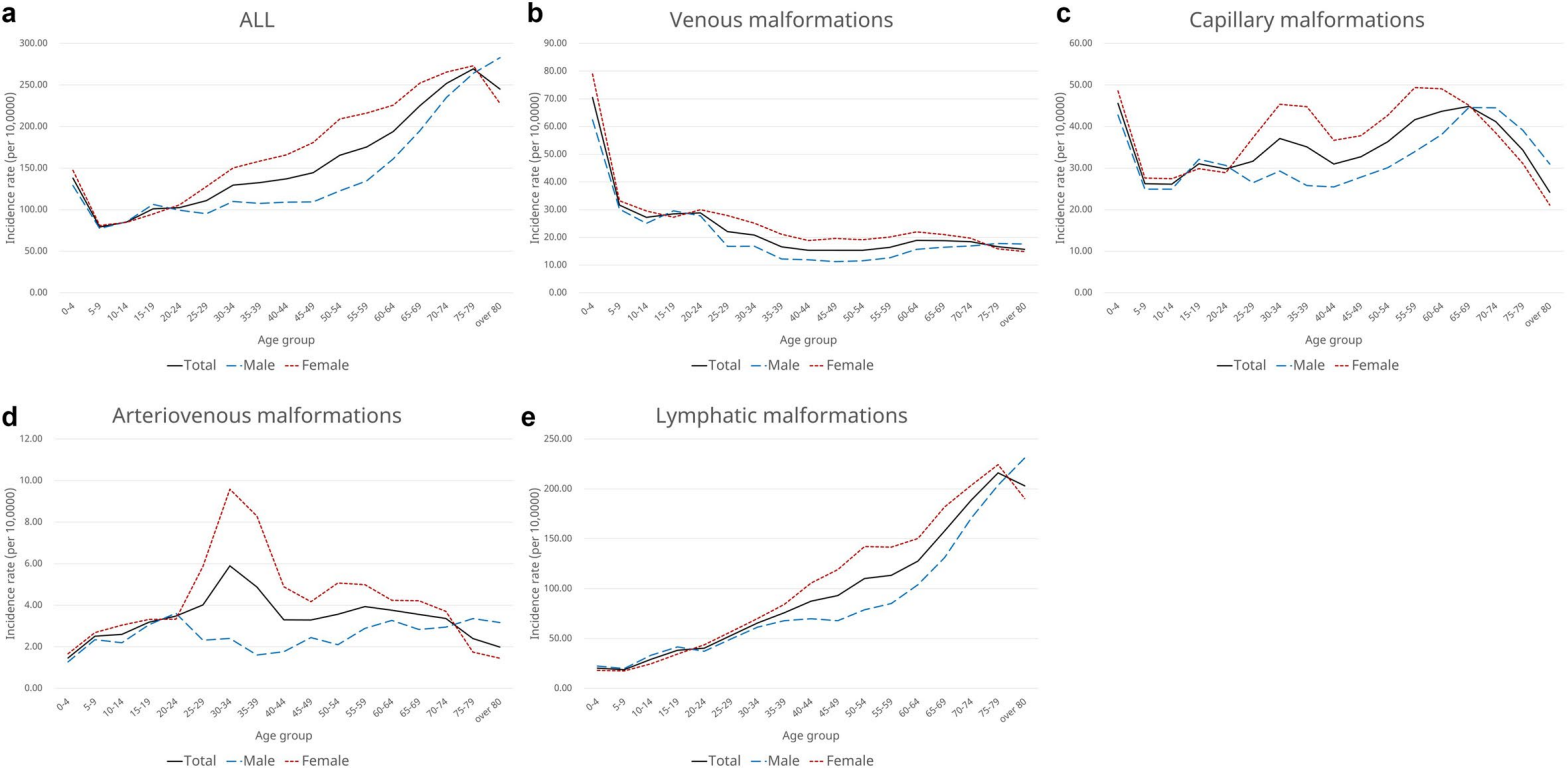
Age-specific incidences of vascular malformations according to subtype. (**a**) Overall vascular malformations, (**b**) venous malformations, (**c**) capillary malformations, (**d**) arteriovenous malformations, (**e**) lymphatic malformations.

In this study, the mean age of participants at the diagnosis of extracranial vascular malformations was 46.14 years, with a range from 32.69 years for VMs to 51.78 years for LMs (Table 1). The age- and sex-specific annual incidences of extracranial vascular malformations are shown in Fig. 2. The highest annual incidence of VMs was observed in female patients aged 0–4 years, with > 70 cases per 100,000 population. Notably, the incidence among both male and female patients tended to decrease with age. The incidence of CMs had three peaks: it was high in female patients aged 0–4, 30–34, and 50–59 years, and in male patients aged 0–4, 15–19, and 65–69 years. The highest incidence was observed in female patients aged 55–59 years. The incidence of AVMs was the highest in male and female patients aged 20–24 and 30–34 years, respectively, and overall, incidence was higher in female patients. The incidence of LMs tended to increase with age in both male and female patients. Before the age of 20 years, the incidence of LMs in male patients was higher than that in female patients; after the age of 20 years, that in female patients was higher than that in male patients.

### Survival and mortality rates

**Figure 3:**
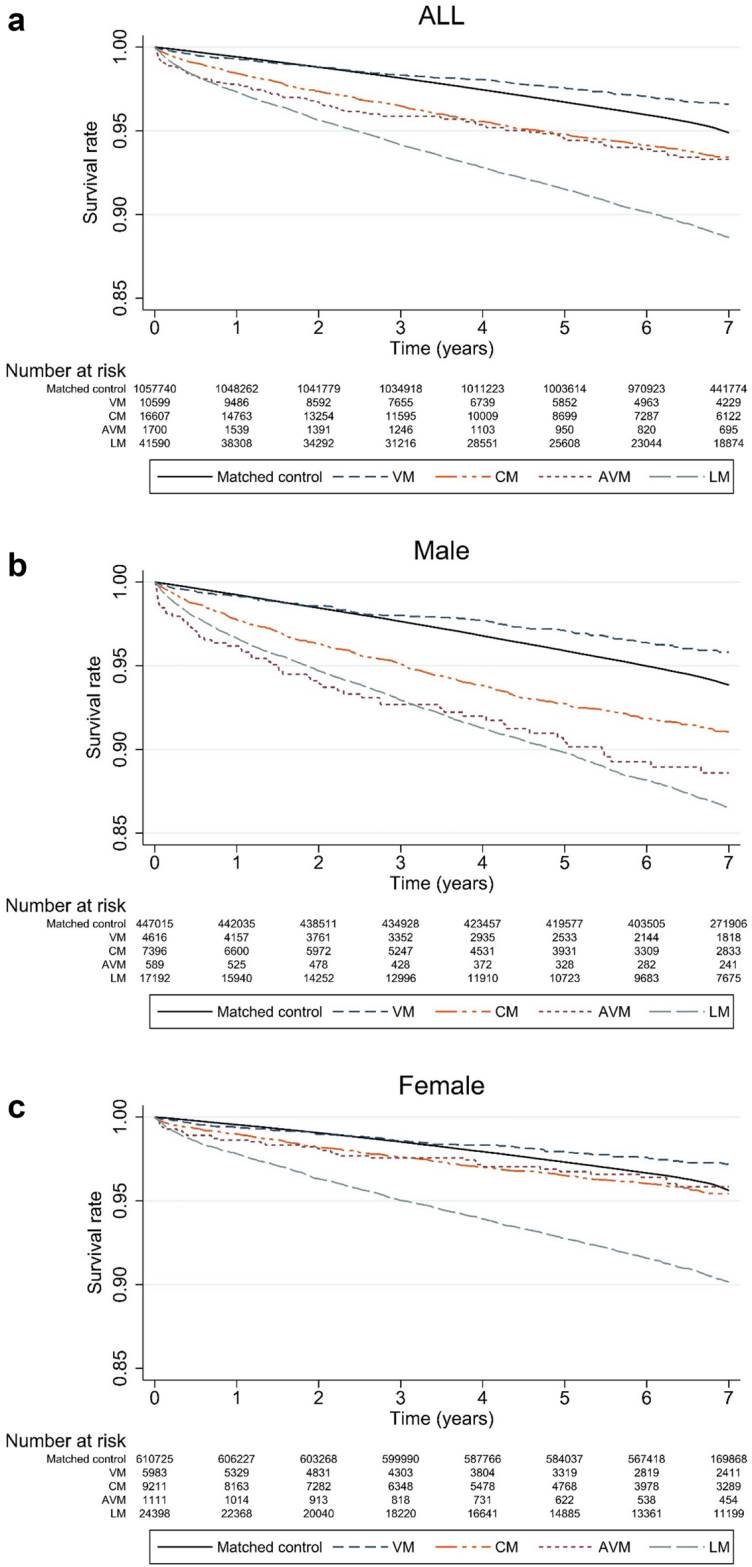
Kaplan–Meier curves for male and female patients with vascular malformations. (**a**) All patients with vascular malformations, (**b**) male patients with vascular malformations, (**c**) female patients with vascular malformations. *VMs* venous malformations, *CMs* capillary malformations, *AVMs* arteriovenous malformations, *LMs* lymphatic malformations.

Overall, 67,736 deaths associated with any cause were recorded for the whole dataset, of which 6306 were in patients with extracranial vascular malformations. The mortality rates per 1000 person-years were 8.67, 5.42, 10.03, 10.33, and 17.05 for the matched controls, those with VMs, those with CMs, those with AVMs, and those with LMs, respectively (Table 3). Patients with extracranial vascular malformations had lower survival rates than those without extracranial vascular malformations, except for VMs. Patients with VMs had slightly lower survival rates than the matched controls for ≤ 2 years following diagnosis; however, they had higher survival rates thereafter (Fig. 3). Cox regression analyses revealed that extracranial vascular malformations, except for VMs, were risk factors for mortality, with HRs of 1.94, 2.06, and 1.38 for CMs, AVMs, and LMs, respectively (Table 4).

**Table 3:** Mortality rates of vascular malformations in Korea from 2008 to 2021 including the matched controls.

	Person-years	Deaths	Mortality rates (per 1000 person-years) (95% CI)
Matched control	7,089,049.70	61,430	8.67 (8.60–8.73)
VMs	64,439.46	349	5.42 (4.88–6.02)
CMs	96,564.51	969	10.03 (9.42–10.69)
AVMs	10,644.57	110	10.33 (8.57–12.46)
LMs	286,104.55	4878	17.05 (16.58–17.53)

*CI* confidence interval, *VMs* venous malformations, *CMs* capillary malformations, *AVMs* arteriovenous malformations, *LMs* lymphatic malformations.

**Table 4:** Cox regression analysis for death of patients with vascular malformations in Korea from 2008 to 2021 (95% CI).

	Hazard ratio	*P* value	95% CI
Birth year	0.90	< 0.001	0.90–0.90
Sex (female)	0.51	< 0.001	0.50–0.52
Vascular malformations			
VMs	1.06	0.278	0.95–1.18
CMs	1.94	< 0.001	1.82–2.07
AVMs	2.06	< 0.001	1.71–2.48
LMs	1.38	< 0.001	1.34–1.42

*CI* confidence interval, *VMs* venous malformations, *CMs* capillary malformations, *AVMs* arteriovenous malformations, *LMs* lymphatic malformations.

## Discussion

This nationwide study identified 70,517 patients with extracranial vascular malformations in Korea from 2008 to 2021, with an overall annual incidence of 9.85 cases per 100,000 population. In the subgroup analysis, the annual incidences of VMs, CMs, AVMs, and LMs during the same period were 1.48, 2.31, 0.24, and 5.82 cases per 100,000 population, respectively. Some studies have reported the incidence of VMs, CMs, and LMs; however, the exact incidence from a comprehensive perspective remains unidentified to date. To the best of our knowledge, the incidence of AVMs has not been reported owing to the rarity of extracranial vascular malformations^30^. In the present study, the incidence of all VMs was higher in female patients, and the malformation subtype with the highest female dominance was AVMs. According to a previous study, CM was found to be the most common vascular malformation^8^; however, in our nationwide study, LM was the most common malformation (5.82 cases per 100,000 population). This difference may be attributed to national or racial differences between the two studies. Furthermore, in our study, the incidence of LMs increased with age; however, relatively older patients may have been underestimated in other studies because they were not well diagnosed given that extracranial vascular malformations are congenital anomalies. In our study, the second most common type of malformation was CM (231 cases per 100,000 population). Further, VMs are known to be the most common type of extracranial vascular malformations for which patients are referred to multidisciplinary vascular anomalies centers^30,31^. However, in the present study, the incidence of VMs was approximately 3.93 times lower than that of LMs, indicating that several patients with LMs are not referred to multidisciplinary vascular anomalies centers. Regarding the age and sex distribution, the incidence of VMs was the highest among female patients aged 0–4 years, indicating that VMs (congenital anomalies) are well diagnosed immediately after birth. Notably, the incidence of CMs peaked immediately after birth, and it was two times higher than that at birth. Moreover, a previous study reported that CMs are present at birth and grow proportionately with the individual^9^. It is possible that the lesion, which was initially small and undiagnosed, may have gradually grown and become diagnosed with age. AVMs are rare and fast-flow malformations, and their exact incidence is currently unknown; however, in the present study, the incidence of AVMs was 0.24 cases per 100,000 population. Among patients with AVMs, male and female patients were most frequently diagnosed in the ages of 20–24 and 30–34 years, respectively, suggesting that AVMs are expressed relatively slower in female patients. Moreover, the history of normal LMs indicates a slow progressive increase in size and complications, including pain, bleeding, and oozing^32^. In the present study, the incidence of LMs tended to increase with age, and this finding is consistent with the known history. Regarding LMs, our nationwide study reported that this diagnosis should not be neglected even in older adults.

Although all pathological mechanisms of extracranial vascular malformations cannot be explained by single gene mutations, VMs, CMs, AVMs, and LMs are known to be related to the Tie-2, GNAQ, MAP2K1, and PIK3CA genes, respectively^1^. VMs are known to be caused by mutations in the Tie-2 gene, which are easily recognizable due to their bluish color and are inferred to be diagnosed at the earliest age among all subtypes. The incidence of CMs and AVMs was highest in women in their 20 s and 30 s, suggesting that related genes may be stimulated by hormones. The activation of the MAP2K1 gene and subsequent production of the MEK1 protein can be triggered by various extracellular signals, including hormones. The binding of a hormone to its specific receptor on the cell surface initiates a cascade of intracellular events that ultimately lead to the activation of the mitogen-activated protein kinase (MAPK) pathway. The PIK3CA gene which is associated with LMs is one of the most commonly mutated genes in human cancers, and mutations in this gene can lead to the dysregulation of the phosphatidylinositol 3-kinase (PI3K) pathway. These mutations can result in increased and sustained activation of the pathway, promoting cell growth and survival and contributing to tumorigenesis and cancer progression. Considering the gradually progressive nature of tumors and cancers, the increasing incidence of LMs with age can be explained.

Extracranial vascular malformations are known as congenital malformations, and the incidence of congenital malformations is generally expressed as the number per 1000 live births^33,34^. Therefore, we initially attempted to express the incidence of these malformations as the number per 1000 births. However, as reported earlier, VMs are mostly diagnosed immediately after birth, but CMs, AVMs, and LMs are not well diagnosed immediately after birth. In particular, in cases of LMs, it might be meaningless to express the number per births because its incidence increases with age. Therefore, we calculated the incidence of extracranial vascular malformations as number per 100,000 of the general population. We believe that there is no definitive approach for classifying types of epidemiological studies, and different classification schemes may be useful for different purposes^35^.

In the present study, all patients with extracranial vascular malformations, except for those with VMs, had higher mortality than the matched controls. Among them, the LM subgroup had the highest mortality (17.05 per 1000 person-years). In addition, based on the Kaplan–Meier curve, patients with LMs had the lowest survival rate as the age of several patients at the diagnosis of LMs was higher than that of patients with other extracranial vascular malformations. Interestingly, the survival rates of patients with AVMs differed in terms of sex. The survival rate of male patients with AVMs was lower than that of the matched controls; moreover, the rate was lower than that of patients with LMs until approximately 3 years following diagnosis. Furthermore, as the incidence of LM in male patients increased with age, the survival rate of male patients with AVM was considered relatively low. In contrast, the survival rate of female patients with AVMs was similar to that of patients with CMs; however, it was higher than that of patients with LMs. Additionally, the survival rate of female patients with AVMs was slightly lower than that of patients with CMs until 3 years following diagnosis, and this rate increased after 3 years. Briefly, the mortality of patients with AVMs was relatively high up to 3 years of diagnosis. Further, the survival rate of patients with VMs was higher than that of matched controls because the incidence of VMs was the highest in individuals aged 0–4-years, which may be attributed to their relatively young age. Age- and sex-adjusted HRs could be obtained in the Cox proportional hazards model, which was used to determine whether the subgroups of extracranial vascular malformations were risk factors for mortality. We found that VM was not a statistically significant risk factor for mortality; however, the adjusted HRs of CMs, AVMs, and LMs were 1.94, 2.06, and 1.38, respectively, indicating that they were risk factors for mortality. Among these, it was confirmed that the HR of AVMs was the highest.

Based on the clinical examination, the Schobinger stage classification categorizes AVMs into stages I–IV according to the severity of symptoms. In stage I, physical examination reveals a warm pink-blue mass, which is occasionally confused with CMs and infantile hemangioma. When this lesion worsens, it enlarges and is accompanied by pulsatility, thrill, and bruit, and this is considered stage II. If the artery-to-vein shunting continues, oxygen diffusion to the capillary does not occur, resulting in an ischemic state, thereby causing secondary pain, tissue ulceration, and bleeding. Moreover, the skin undergoes dystrophic changes, and this is considered stage III. Notably, AVMs allow direct blood flow through the nidus from the high-resistance high-pressure arterial system to the low-resistance low-pressure venous system without capillary perfusion, leading to venous hypertension. When this becomes severe, it leads to high-output cardiac failure corresponding to Schobinger stage IV (Table 5)^36^. The low survival rate and the highest adjusted HR of AVMs among the vascular malformation subgroups, despite the incidence of AVMs being the highest in the 20 s and 30 s, can be attributed to the fact that they can cause cardiovascular disease. Kim et al. suggested that patients with AVM had to bear higher hospital costs owing to the complexity of their condition, which requires a higher level of inpatient care^37^. This report can be evidence of the high mortality of AVMs.

**Table 5:** Schobinger staging of arteriovenous malformations.

Stage	Clinical findings
I (Quiescence)	Warm, pink-blue, shunting on Doppler ultrasonography
II (Expansion)	Enlargement, pulsation, thrill, bruit, and tortuous veins
III (Destruction)	Dystrophic skin changes, ulceration, bleeding, and pain
IV (Decompensation)	Cardiac failure

Regarding the mortality of LMs, because they are associated with mutations in the PIK3CA gene, and the PIK3CA gene is associated with several cancers, patients with LMs may be more likely to develop cancer. In such a case, it is possible to explain the higher mortality rate of LMs patients than control cohort.

This study has some limitations. First, as the ICD-10 code was used to identify the diagnostic classification, the presence of combined types of extracranial vascular malformations (including lymphatico-venous malformations and CM-AVM) was impossible. Second, there was no information on the findings of imaging tests, including MRI to diagnose VM or LM and computed tomography angiogram to diagnose AVM, or photographs derived from these data. Thus, there may be a bias in the classification of diagnoses. However, the Korean NHIS provides fairly accurate diagnostic data. Before claiming the medical fee, the ICD-10 code and fees for surgery and intervention were verified by the insurance review team of each general hospital. Subsequently, the Korean NHIS data were stored after reverification by the Health Insurance Review and Assessment service—a Korean government agency^25^. Further, we evaluated the diagnostic accuracy of diagnostic classification, which revealed high sensitivity and specificity. Therefore, the risk of bias for misdiagnosis in the present study was low. Third, regarding mortality, it would have been more useful to determine the causes of death in patients with extracranial vascular malformations and in matched controls. In NHIS-NHID–based studies, the database did not contain data about the causes of death. In contrast, Statics Korea has information on the causes of deaths, and we have not conducted a study that linked the NHIS-NHID and Statics Korea. However, as data of all patients with extracranial vascular malformations were extracted and those of matched controls were extracted by age and sex from the general populations, no selection bias was noted between patients with extracranial vascular malformations and matched controls in this study. Therefore, the only difference in the cause of death between the two groups was attributed to extracranial vascular malformations.

This study also has several strengths. First, to the best of our knowledge, this is the first study to investigate the incidence of extracranial vascular malformations and related mortality in a nationally representative Asian population. Previous studies investigated hospital-based data or used small sample sizes, posing difficulties in the identification of the incidence of rare extracranial vascular malformations, including AVMs. Second, by evaluating the incidence in terms of age and sex, were identified the age and sex that demonstrated the highest incidence of each vascular malformation subtype. Finally, the survival and mortality of the patients in each subgroup with extracranial vascular malformations were identified by comparison with the nationally matched control population.

## Conclusion

This study revealed that the overall annual incidence of extracranial vascular malformations was 9.85 cases per 100,000 population in Korea from 2008 to 2021. Among the vascular malformation subgroups, the proportion of LMs was the highest, whereas that of AVMs was the lowest. Compared with the mortality of the matched general population, the mortality of patients with extracranial vascular malformations was high, except for those with VMs. The adjusted HR for the mortality of AVMs was the highest among the vascular malformation subgroups. Therefore, clinicians in vascular anomalies clinics need to take care of patients with a high risk of mortality due to extracranial vascular malformations, except for VMs.

## Data Availability

The data that supports the findings of this study is available from the NHI service, but restrictions apply to the availability of data, which was used with permission for the current study and therefore not publicly available. Data is however available upon reasonable request and with permission of NHI. The derived data generated in this research will be shared on reasonable request to the corresponding author.
